# Influence of frailty on short-term mortality in older patients with multiple trauma in the emergency department

**DOI:** 10.1007/s00068-026-03214-4

**Published:** 2026-06-11

**Authors:** Şimşek Çelik, Abdulkerim Toker, Süleyman Biçer, İsmail Kıvanç Cebecioğlu

**Affiliations:** Department of Emergency Medicine, Sivas Cumhuriyet University Faculty of Medicine, Sivas, Türkiye

**Keywords:** Emergency department, Older patient, Polytrauma, Frailty, PRISMA-7, Prognosis

## Abstract

**Purpose:**

To investigate the association between frailty and 28-day mortality in older patients presenting to the emergency department with multiple trauma and to assess the discriminative performance of PRISMA-7 and the Trauma-Specific Frailty Index (TSFI) in frailty assessment in relation to commonly used trauma severity scores.

**Methods:**

This prospective observational study included patients aged ≥ 65 years with multiple trauma admitted to the emergency department between July 1 and August 31, 2024. Frailty was assessed using PRISMA-7 and TSFI. Patients were stratified into frail and non-frail groups and further categorized according to age groups (65–74, 75–84, and ≥ 85 years). Clinical characteristics, trauma severity scores, and outcomes, including 28-day mortality, were compared. Receiver operating characteristic (ROC) analysis was used to evaluate discriminative performance, and logistic regression analysis was performed to assess the association between scores and mortality.

**Results:**

A total of 144 patients were included. Frailty was significantly associated with increased 28-day mortality, higher ICU admission rates, and longer hospital stays (*p* < 0.001). Mortality increased with age, reaching 30.0% in patients aged ≥ 85 years (*p* = 0.006). Frailty scores (PRISMA-7 and TSFI) increased significantly with age, whereas ISS did not differ significantly across age groups. ROC analysis demonstrated comparable discriminative performance among scoring systems, with PRISMA-7 showing balanced sensitivity and specificity. In logistic regression analysis, all scores were significantly associated with mortality, and frailty identified by PRISMA-7 was associated with a higher mortality risk.

**Conclusion:**

Frailty is significantly associated with adverse outcomes, including increased mortality, in older trauma patients. Frailty assessment using simple screening tools such as PRISMA-7 may complement traditional trauma severity scores and help identify high-risk patients in emergency settings.

**Supplementary Information:**

The online version contains supplementary material available at 10.1007/s00068-026-03214-4.

## Introduction

United Nations projections indicate a substantial increase in the global population of older adults. According to these projections, the number of individuals aged 80 years and older is expected to rise from 137 million in 2017 to 425 million in 2050 and approximately 909 million in 2100 [1]. As the older population grows, trauma in older adults is becoming an increasingly important public health concern. Individuals aged 65 years and older account for a considerable proportion of trauma cases, and trauma has been reported as one of the leading causes of death in this age group in several healthcare systems [2]. The increasing life expectancy, adoption of more active lifestyles among older individuals, and physiological changes associated with aging contribute to a higher susceptibility to traumatic injuries [3, 4].

Falls, motor vehicle accidents, and other traumatic events represent major causes of morbidity and mortality in older adults. Despite similar injury severity, older trauma patients frequently experience worse clinical outcomes compared with younger individuals [4, 5]. One of the major factors contributing to these unfavorable outcomes is frailty, a multidimensional geriatric syndrome characterized by decreased physiological reserve, impaired homeostatic mechanisms, and reduced resilience to external stressors [5].

Frailty has been increasingly recognized as an important determinant of outcomes in older trauma patients. Chronological age alone does not adequately explain variations in trauma outcomes, as factors such as comorbidities, functional status, cognitive impairment, and nutritional status differ substantially among individuals of the same age group. Consequently, older trauma patients represent a heterogeneous population ranging from independent individuals to those with significant frailty and limited physiological reserve. Previous studies have shown that frailty is associated with increased complications, longer hospital stays, higher intensive care requirements, and increased mortality among trauma patients [6].

Several screening tools have been developed to assess frailty in clinical settings. Among these, the Trauma-Specific Frailty Index (TSFI) and the Program of Research to Integrate Services for the Maintenance of Autonomy 7 (PRISMA-7) are commonly used instruments designed to identify frailty in older adults [2, 7]. PRISMA-7 is a simple seven-item screening tool that can be rapidly applied in clinical practice, while TSFI is a multidimensional index specifically developed for trauma populations. Although these instruments are primarily designed to identify frailty rather than directly predict mortality, frailty itself has been shown to be associated with adverse outcomes in older patients.

Despite growing recognition of the clinical importance of frailty, its systematic assessment in emergency departments remains limited. Previous studies have reported that frailty assessment is performed in only a small proportion of older trauma patients in some centers [5]. Failure to recognize frailty early may contribute to delays in risk stratification and appropriate care planning in this vulnerable population.

Therefore, early identification of frailty in older trauma patients may play an important role in improving clinical decision-making and optimizing patient management strategies in emergency departments. The aim of this study was to investigate the association between frailty and 28-day mortality in older patients presenting to the emergency department with multiple trauma and to assess the discriminative performance of PRISMA-7 and the Trauma-Specific Frailty Index (TSFI) in frailty assessment in relation to commonly used trauma severity scores such as the Glasgow Coma Scale (GCS), Revised Trauma Score (RTS), and Injury Severity Score (ISS).

## Materials and methods

### Study design and setting

This study is a prospective, observational study. It was approved by the Ethics Committee (Date: 27/06/2024; No: 2024/06–33). The study was conducted in accordance with the principles of the Declaration of Helsinki. Written informed consent was obtained from all patients or their legal representatives.

### Selection of participants

All patients aged 65 years and older who presented to the emergency department of Sivas Cumhuriyet University Medical Faculty Hospital between July 1 and August 31, 2024 were screened for eligibility. In this study, older adults were defined as individuals aged 65 years and above. Multiple trauma was defined as the presence of injuries involving two or more body regions. Consecutive eligible patients meeting these criteria were included in the study.

### Inclusion and exclusion criteria

All patients aged 65 years and older with multiple trauma who agreed to participate in this study were included. Patients with isolated minor injuries limited to a single body region and those with incomplete or missing data were excluded from the study. Data were collected by a dedicated study team composed of medical doctors who were not involved in the clinical management of the patients and were blinded to the study hypothesis.

Frailty was assessed using PRISMA-7 (Appendix 1) and the Trauma-Specific Frailty Index (TSFI) (Appendix 2). Although PRISMA-7 was originally validated for individuals aged ≥ 75 years, it has also been applied in studies involving adults aged ≥ 65 years to screen for frailty in emergency settings. Prior to the study, all members of the study team received a 30-minute training session on the basic concepts of frailty and the application of the assessment tools.

Demographic and clinical data, including age, sex, mechanism of trauma, PRISMA-7 and TSFI scores, Glasgow Coma Scale (GCS), Revised Trauma Score (RTS), Injury Severity Score (ISS), and the patient’s final status in the emergency department, were obtained from the study questionnaire and hospital medical records. These scoring systems are routinely recorded for all older trauma patients admitted to the emergency department.

### Outcome measures

Patients were evaluated in two ways. First, frailty status was determined using PRISMA-7 and the Trauma-Specific Frailty Index (TSFI), and patients were stratified into frail and non-frail groups according to the respective cut-off values. These groups were compared in terms of demographic characteristics, mechanism of trauma, 28-day mortality, Intensive Care Unit (ICU) admission, length of hospital stay, and clinical scores including PRISMA-7, TSFI, Glasgow Coma Scale (GCS), Revised Trauma Score (RTS), and Injury Severity Score (ISS).

Second, patients were categorized as survivors and non-survivors based on 28-day mortality, and the same parameters were compared between the two groups. The date of death was obtained from electronic health records, official records, insurance data, or by contacting the patients’ relatives using the telephone numbers registered in the hospital electronic system. In addition, patients were stratified into three age groups (65–74 years, 75–84 years, and ≥ 85 years), and demographic characteristics, clinical parameters, and outcomes were compared across these groups.

The optimal cut-off values for PRISMA-7 and TSFI were determined using receiver operating characteristic (ROC) curve analysis based on 28-day mortality. According to this analysis, the optimal cut-off value for PRISMA-7 was identified as 4.5. Patients with PRISMA-7 scores < 4.5 were classified as less frail, whereas those with scores ≥ 4.5 were classified as more frail. Similarly, the optimal cut-off value for TSFI was determined as 0.30, and patients were categorized as less frail (< 0.30) and more frail (≥ 0.30).

### Statistical analysis

The required sample size was calculated using G*Power software (version 3.1.6) based on data from previous studies [8–10]. A minimum sample size of 144 patients was estimated to detect significant differences between groups with a statistical power of 95% and a type I error rate (α) of 5%.

Statistical analyses were performed using SPSS software version 26.0 (IBM Corp., Armonk, NY, USA). Continuous variables were assessed for normality and presented as medians with interquartile ranges (IQR), while categorical variables were expressed as numbers and percentages. Comparisons between groups were performed using the Mann–Whitney U test for continuous variables and the chi-square (χ²) test for categorical variables. For comparisons across more than two groups (age stratification), the Kruskal–Wallis test was used for continuous variables.

Binary logistic regression analysis was performed to evaluate the association between frailty scores and 28-day mortality in older trauma patients. Receiver operating characteristic (ROC) curve analysis was used to evaluate the discriminative performance of the scoring systems for 28-day mortality. The area under the curve (AUC) with 95% confidence intervals (CI) was calculated for each score. The optimal cut-off values were determined using the Youden index. P-value of < 0.05 was considered statistically significant.

## Results

**Fig. 1:**
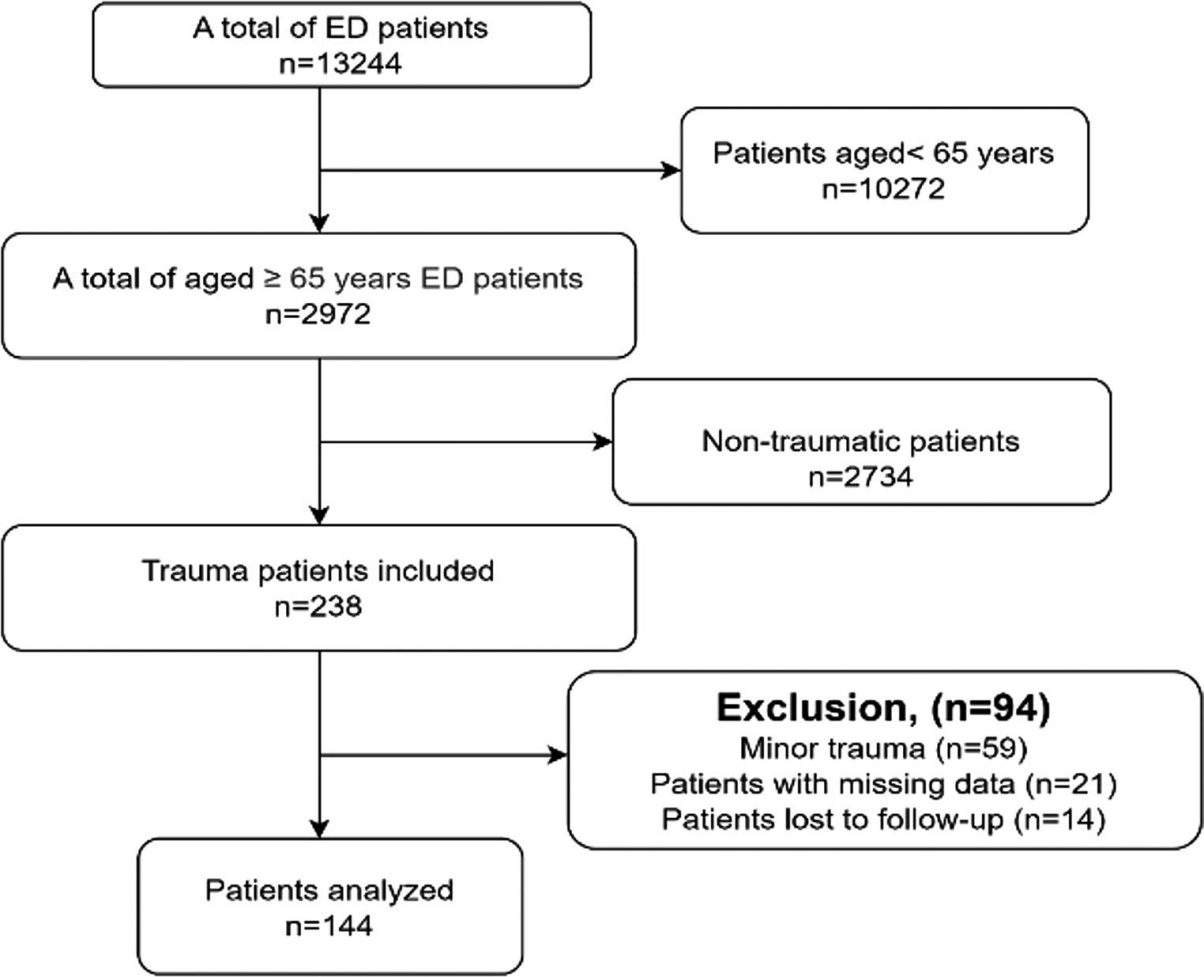
Flow chart of patient

Table 1 shows the characteristics and demographic features of fragile, non-fragile, and all patients according to PRISMA-7 and TSFI. Of the 238 eligible patients encountered during our study period, 144 older trauma patients who met the criteria were included in the study (Fig. 1; Table 1). The median age of participants was 74 years (interquartile range, 70–80 years), and 46.5% (*n* = 67) were female (Table 1). Mechanical fall constituted the most common trauma mechanism in 82.6% (*n* = 119) of cases. The 28-day mortality rate, ICU admission rate, and mean length of hospital stay were 7.6% (*n* = 11), 6.2% (*n* = 9), and 2 days (interquartile range: 0–7 days), respectively.

Both PRISMA-7 and TSFI scores showed that frail patients were statistically significantly older (*p* = 0.015 and *p* = 0.019, respectively), but gender did not significantly affect frailty in either score (*p* = 0.311 and *p* = 0.998, respectively). Of the patients, 82.6% (*n* = 119) presented to the emergency department as a result of a mechanical fall. In terms of the mechanism of trauma, the difference between the groups was statistically significant according to the TSFI (*p* = 0.031), but not according to the PRISMA-7 (*p* = 0.735). In both PRISMA-7 and TSFI, the 28-day mortality rate, ICU admission rate, and average length of hospital stay increased with increasing frailty and were found to be statistically significant (*p* < 0.001). In both scales, GCS and RTS were significantly lower in frail patients, while ISS, TSFI, and PRISMA-7 were significantly higher (*p* < 0.005) (Table 1).

**Table 1:** Clinical characteristics of frail and non-frail groups according to PRISMA-7 and TSFI

Variables		PRISMA-7			TSFI		
	Total (*n* = 144)	non-Frail (*n* = 106)	Frail (*n* = 38)	*P* value	non-Frail (*n* = 101)	Frail (*n* = 43)	*P* value
**Age**,** years**	74 (70–80)	73 (69–78)	78 (74–84)	0.015	73 (69–79)	77 (73–82)	0.019
**Female sex**,** n (%)**	67 (46.5)	52 (49.1)	15 (39.5)	0.311	47 (46.5)	20 (46.5)	0.998
**Trauma mechanism**,** n (%)**							
Mechanical fall	119 (82.6)	87 (82.1)	32 (84.2)	0.735	79 (78.2)	40 (93.0)	0.031
Motor vehicle accident	23 (16.0)	17 (16.0)	6 (15.8)		20 (19.8)	3 (7.0)	
Penetrating injury	2 (1.4)	2 (1.9)	0 (0)		2 (2.0)	0 (0)	
**28-day mortality**,** n (%)**	11 (7.6)	2 (1.9)	9 (23.7)	< 0.001	3 (3.0)	8 (18.6)	< 0.001
**ICU admission**,** n (%)**	9 (6.2)	2 (1.9)	7 (18.4)	< 0.001	2 (2.0)	7 (16.3)	< 0.001
**Length of hospital stay**,** days**	2 (0–7)	0 (0–5)	7.5 (3–10)	< 0.001	0 (0–5)	5 (0–10)	< 0.001
**SCORE**							
GCS	15 (15–15)	15 (15–15)	14.5 (13–15)	< 0.001	15 (15–15)	15 (13–15)	0.002
RTS	12 (11–12)	12 (11–12)	11 (10–12)	0.005	12 (11–12)	11 (11–12)	0.039
ISS	9 (6–11)	6 (3-9.5)	11 (6-14.25)	0.003	6 (3–10)	9 (6–14)	0.015
TSFI	0.20 (0.13–0.36)	0.16 (0.12–0.26)	0.45 (0.33–0.53)	< 0.001	0.16 (0.10–0.21)	0.51(0.36–0.55)	< 0.001
PRISMA-7	2 (1–5)	2 (1–3)	5 (5–6)	< 0.001	2 (1–3)	5 (4–6)	< 0.001

The data are expressed as the medians (IQR) or number (%) of patients. Comparisons between groups were performed using Mann-Whitney U tests for continuous variables and χ2 tests for categorical data. PRISMA-7, the 7-question tool of the Program on Research for Integrating Services of the Maintenance of Autonomy; TSFI, Trauma-Specific Frailty Index; GCS, Glasgow Coma Scale; RTS, RevisedTraumaScore; ISS, Injury Severity Score; ICU, Intensive care unit

When patients were stratified into three age groups (65–74 years, 75–84 years, and ≥ 85 years), significant differences were observed across several clinical parameters. As expected, median age differed significantly between the groups (*p* < 0.001). There was no statistically significant difference in sex distribution or trauma mechanism among the age groups (*p* = 0.580 and *p* = 0.226, respectively). A significant increase in 28-day mortality was observed with advancing age, rising from 2.6% in the 65–74 age group to 30.0% in patients aged ≥ 85 years (*p* = 0.006). Although ICU admission rates were higher in the oldest group, this difference did not reach statistical significance (*p* = 0.136). Similarly, length of hospital stay increased significantly with age, with the longest median duration observed in patients aged ≥ 85 years (*p* < 0.001). Regarding clinical scores, significant differences were observed across age groups for GCS (*p* < 0.001), RTS (*p* = 0.001), TSFI (*p* < 0.001), and PRISMA-7 (*p* < 0.001). Frailty scores, including TSFI and PRISMA-7, showed a clear increasing trend with age, indicating a higher level of frailty in older patients. ISS scores were higher in older age groups; however, this difference was not statistically significant (*p* = 0.052). Overall, these findings suggest that increasing age is associated with higher frailty levels, longer hospital stays, and increased short-term mortality, while trauma severity scores alone may not fully capture this risk (Table 2).

**Table 2:** Patient characteristics stratified by age group

Variables	Total (*n* = 144)	65–74 years (*n* = 76)	75–84 years (*n* = 58)	≥ 85 years (*n* = 10)	*P* value
**Age**,** years**	74 (70–80)	70 (68–72)	79 (76–81)	87 (86–93)	< 0.001
**Female Sex**,** n (%)**	67 (46.5)	33 (43.4)	30 (51.7)	4 (40.0)	0.580
**Trauma mechanism, n (%)**					
Mechanical fall Motor vehicle accident Penetrating injury	119 (82.6) 23 (16.0) 2 (1.4)	59 (77.6) 15 (19.7) 2 (2.6)	51 (87.9) 7 (12.1) 0 (0)	9 (90.0) 1 (10.0) 0 (0)	0.226
**28-day mortality**,** n (%)**	11 (7.6)	2 (2.6)	6 (10.3)	3 (30.0)	0.006
**ICU admission**,** n (%)**	9 (6.2)	5 (6.6)	2 (3.4)	2 (20.0)	0.136
**Length of hospital stay**,** days**	2 (0–7)	0 (0–5)	3.5 (0-8.25)	10 (6.75–12.25)	< 0.001
**SCORE**					
GCS RTS ISS TSFI PRISMA-7	15 (15–15) 12 (11–12) 9 (6–11) 0.20 (0.13–0.36) 2 (1–5)	15 (15–15) 12 (11–12) 6 (3–11) 0.16 (0.10–0.26) 2 (1–3)	15 (15–15) 11 (11–12) 9 (6–11) 0.28 (0.20–0.36) 3 (1.75-5)	13 (13–15) 10.5 (9–12) 10 (9–13) 0.46 (0.34–0.52) 5 (4–6)	< 0.001 0.001 0.052 < 0.001 < 0.001

The data are expressed as the medians (IQR) or number (%) of patients. Comparisons between groups were performed using Kruskal–Wallis test for continuous variables and χ2 tests for categorical data. PRISMA-7, the 7-question tool of the Program on Research for Integrating Services of the Maintenance of Autonomy; TSFI, Trauma-Specific Frailty Index; GCS, Glasgow Coma Scale; RTS, Revised Trauma Score; ISS, Injury Severity Score; ICU, Intensive care unit

The demographic characteristics and features of patients according to their mortality status are presented in Table 3. The ages of patients who did not survive were found to be significantly higher (*p* = 0.002). 9.1% (*n* = 1) of patients who died were female. A statistically significant difference was found between the groups in terms of mortality and gender (*p* = 0.010). In terms of trauma mechanism, no statistically significant difference was found between the groups (*p* = 0.904). The admission rate to the ICU and the average length of hospital stay were increased in older patients with a fatal outcome and were found to be statistically significant (*p* < 0.001). In non-survival patients, GCS and RTS were significantly lower, while ISS, TSFI, and PRISMA-7 were significantly higher (*p* < 0.001) (Table 3).

**Table 3:** Comparison of patient groups according to 28-day mortality

Variables	Survival (*n* = 133)	Non-survival (*n* = 11)	*P* value
**Age**,** years**	74 (69–79)	78 (75–88)	0.002
**Female sex**,** n (%)**	66 (49.6)	1 (9.1)	0.010
**Trauma mechanism**,** n (%)**			
Mechanical fall	110 (82.7)	9 (81.8)	0.904
Motor vehicle accident	21 (15.8)	2 (18.2)	
Penetrating injury	2 (1.5)	0 (0)	
**ICU admission**,** n (%)**	3 (2.3)	6 (54.5)	< 0.001
**Length of hospital stay**,** days**	2 (0–6)	10 (8–12)	< 0.001
**SCORE**			
GCS	15 (15–15)	13 (11–14)	< 0.001
RTS	12 (11–12)	10 (8–11)	< 0.001
ISS	6 (6–11)	11 (9–22)	< 0.001
TSFI	0.20 (0.13–0.33)	0.53 (0.36–0.63)	< 0.001
PRISMA-7	2 (1–4)	5 (5–5)	< 0.001

The data are expressed as the medians (IQR) or number (%) of patients. Comparisons between groups were performed using Mann-Whitney U tests for continuous variables and χ2 tests for categorical data. PRISMA-7, the 7-question tool of the Program on Research for Integrating Services of the Maintenance of Autonomy; TSFI, Trauma-Specific Frailty Index; GCS, Glasgow Coma Scale; RTS, Revised Trauma Score; ISS, Injury Severity Score; ICU, Intensive care unit

**Fig. 2:**
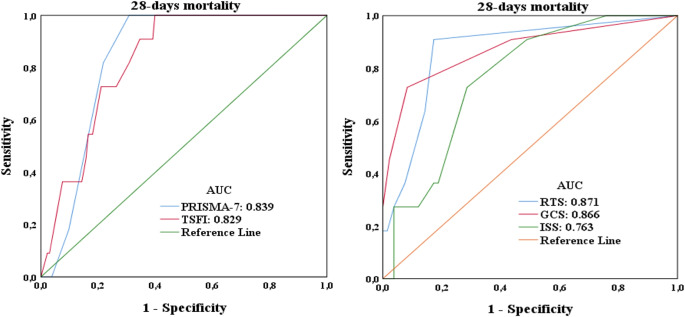
Receiver operating characteristic (ROC) curve of 28-day mortality prediction. PRISMA-7, the 7-question tool of the Program on Research for Integrating Services of the Maintenance of Autonomy; TSFI, Trauma-Specific Frailty Index; GCS, Glasgow Coma Scale; RTS, Revised Trauma Score; ISS, Injury Severity Score

The performance of RTS, GCS, PRISMA-7, TSFI, and ISS scores in relation to 28-day mortality is shown (Fig. 2). For 28-day mortality, the area under the ROC curve was 0.871 (95% CI: 0.740-1.000), 0.866 (95% CI: 0.759–0.974), 0.839 (95% CI: 0.773–0.906), 0.829 (95% CI: 0.744–0.914), and 0.763 (95% CI: 0.647–0.879), respectively.

The cut-off value for RTS was 10.5, for GCS was 14.0, for PRISMA-7 was 4.5, for TSFI was 0.3, and for ISS was 10. The sensitivity and specificity of RTS were 72.7% and 91.7%, respectively; the sensitivity and specificity of GCS were 90.9% and 82.7%, respectively; The sensitivity and specificity of PRISMA-7 were 81.8% and 78.2%, respectively; the sensitivity and specificity of TSFI were 72.7% and 73.7%, respectively; the sensitivity and specificity of ISS were 72.7% and 71.4%, respectively. All parameters were found to be statistically significant (*p* < 0.001).

For mortality, the areas under the ROC curve were compared in pairs, and no significant difference was found between the groups (*p* > 0.005). The prevalence of frailty (PRISMA-7 score > 4.5) in older multitrauma patients in the emergency department using PRISMA-7 (cut-off value = 4.5) was 38 (26.4%) patients. The prevalence of frailty (TSFI score > 0.30) among older multitrauma patients in the emergency department using TSFI (cut-off value = 0.30) was 43 (29.9%) patients.

Logistic regression analysis was performed using 28-day mortality as the dependent variable (Y = 1 for the death group and Y = 0 for the survival group) and five scores as independent variables. The results are presented in Table 4. PRISMA-7, TSFI, GCS, RTS, and ISS were significantly effective factors in determining the 28-day mortality risk of older trauma patients.

**Table 4:** Logistic regression analysis of the association between scoring systems and 28-day mortality in older trauma patients

Factors	Coefficient of Regression	Standard Error	Wald	*P*	OR (95% CI)
PRISMA-7	1.16	0.21	27.14	< 0.001	3.36(2.06–4.85)
TSFI	0.67	0.18	6.13	0.004	1.94(1.87–29.73)
GCS	0.82	0.14	12.17	< 0.001	2.12(1.52–4.44)
RTS	0.63	0.17	13.52	< 0.001	1.89(1.34–2.66)
ISS	0.44	0.13	10.74	0.001	1.56(1.19–2.04)

OR, odds ratio; PRISMA-7, the 7-question tool of the Program on Research for Integrating Services of the Maintenance of Autonomy; TSFI, Trauma-Specific Frailty Index; GCS, Glasgow Coma Scale; RTS, Revised Trauma Score; ISS, Injury Severity Score

## Discussion

Older trauma patients frequently present to emergency departments with complex and potentially life-threatening conditions. In this population, accurate risk assessment is essential for guiding clinical monitoring, treatment strategies, and resource allocation. Frailty has been increasingly recognized as an important factor influencing outcomes in older trauma patients. Due to reduced physiological reserve and impaired stress response, frail individuals may experience severe consequences even after relatively minor traumatic events. Therefore, in addition to assessing trauma severity, evaluating frailty may provide valuable information in identifying patients at increased risk of adverse outcomes. In this study, we investigated the relationship between frailty and clinical outcomes, including 28-day mortality, in older trauma patients presenting to the emergency department.

Our findings demonstrated that frailty in older trauma patients was significantly associated with increased 28-day mortality, higher intensive care unit (ICU) admission rates, and longer hospital stays. These results are consistent with previous studies and recent guidelines indicating that frailty is an important determinant of outcomes in older trauma patients [3, 4, 6]. Previous research has shown that frail patients have higher in-hospital and short-term mortality rates even when injury severity is relatively low, and that frailty is also associated with increased ICU admission and prolonged hospitalization [3, 6, 11, 12]. These findings highlight the importance of evaluating frailty in addition to conventional trauma severity indicators.

In the present study, commonly used trauma severity scores such as RTS, ISS, and GCS, as well as frailty assessment tools including TSFI and PRISMA-7, showed significant differences between survivors and non-survivors. Previous studies have similarly reported that trauma scores such as RTS, ISS, and GCS are associated with mortality in trauma patients, while frailty assessment tools such as TSFI and PRISMA-7 have been shown to be related to adverse outcomes in older adults [13–18]. In our ROC analysis, the discriminative performance of PRISMA-7 for 28-day mortality was lower than that of RTS and GCS but higher than that of TSFI and ISS. However, when all parameters were compared in pairwise analyses, no statistically significant differences were observed between the scoring systems. These findings suggest that both traditional trauma scores and frailty assessment tools provide useful but comparable information in evaluating outcomes in older trauma patients.

Our study also demonstrated that when the PRISMA-7 cut-off value determined by ROC analysis was set at 4.5, the tool showed balanced sensitivity and specificity values of approximately 80% for identifying patients at higher risk of mortality. Although PRISMA-7 was originally developed as a screening tool for frailty rather than a direct predictor of mortality, our findings indicate that frailty identified by this tool is associated with increased mortality risk in older trauma patients. This supports the concept that frailty assessment may complement traditional trauma severity scores in identifying vulnerable patients in emergency settings.

In addition, when patients were stratified according to age groups, a clear trend toward increased mortality, longer hospital stay, and higher frailty scores was observed with advancing age. Notably, although trauma severity scores such as ISS did not differ significantly across age groups, mortality rates increased substantially in the oldest patients. This finding suggests that factors beyond anatomical injury severity, particularly frailty and reduced physiological reserve, play a critical role in determining outcomes in older trauma patients. These results support the concept that chronological age alone is insufficient to explain clinical outcomes and highlight the importance of incorporating frailty assessment into routine evaluation of older trauma patients in emergency settings.

Regression analysis in our study showed that PRISMA-7, TSFI, GCS, RTS, and ISS were significantly associated with 28-day mortality in older trauma patients. Among these variables, patients classified as frail according to PRISMA-7 had a 3.36-fold higher risk of mortality compared with less frail individuals. This finding is consistent with previous studies reporting that frailty is an independent risk factor for mortality in older trauma patients [6, 19]. Reduced physiological reserve, impaired stress response, and the presence of multiple comorbidities may explain the increased vulnerability of frail individuals even when injury severity appears limited.

In addition to its clinical relevance, PRISMA-7 may offer practical advantages in emergency department settings. Compared with TSFI, which requires more detailed information regarding physical activity and social history, PRISMA-7 is a brief and easily applicable screening tool that can be rapidly administered in acute care settings. Previous studies have reported that PRISMA-7 has higher completion rates and better feasibility in emergency departments compared with more complex frailty assessment instruments [5]. In this respect, PRISMA-7 may be particularly useful as an initial screening tool to identify frail older trauma patients who may require closer monitoring and more comprehensive geriatric assessment.

### Strengths and limitations

This study has several strengths and limitations. To our knowledge, this study is one of the first to assess frailty in older trauma patients using PRISMA-7 and to examine its association with mortality and clinical outcomes, thereby contributing to the existing literature. However, several limitations should be acknowledged. First, the study was conducted in a single center with a relatively homogeneous and limited sample size, which may restrict the generalizability of the findings. Second, the study period was relatively short and limited to a specific seasonal interval (July 1 to August 31), during which trauma cases may be more frequent; therefore, the results may not fully reflect variations across the entire year. Third, although frailty was assessed using validated tools, other important geriatric parameters such as cognitive status, functional dependency, and nutritional status were not evaluated. In addition, older patients often have multiple comorbidities that may influence clinical outcomes. Finally, patients were evaluated during the initial emergency department assessment, and outcomes were based on 28-day mortality without classification according to final diagnoses, which may have introduced variability in the results.

## Conclusion

In older trauma patients presenting to the emergency department, PRISMA-7 appears to be a reliable and practical tool for assessing frailty. Frailty identified by PRISMA-7 was significantly associated with increased 28-day mortality and adverse clinical outcomes. These findings suggest that frailty assessment using PRISMA-7 may provide additional and complementary information beyond traditional trauma severity scores. Therefore, incorporating simple frailty screening tools such as PRISMA-7 into routine clinical practice may improve early risk stratification and clinical decision-making in emergency settings. However, further large-scale and multicenter studies are needed to validate these findings.

## Supplementary Information

Below is the link to the electronic supplementary material.

Supplementary Material 1

Supplementary Material 2

## Data Availability

The datasets used for analysis in this study are available from the corresponding author upon reasonable request.
