# Supplementary material for: Influence of frailty on short-term mortality in older patients with multiple trauma in the emergency department
Source: Eur J Trauma Emerg Surg. 2026 Jun 11;52(1):185. doi: 10.1007/s00068-026-03214-4 (PMC13260031; doi:10.1007/s00068-026-03214-4)
Supplement: Supplementary file 1 — Supplementary Material 1 [file 68_2026_3214_MOESM1_ESM.pdf]

Appendix 1.The PRISMA-7 tool

Questions	Answer		score
1.Are you more than 85 years old?	yes	no	1
2.Male?	yes	no	1
3.In general,do you have any health problems that require you to limit your actvities?	yes	no	1
4.Do you need someone to help you on a regular basis	yes	no	1
5.In general,do you have any health problems that require you to stay at home?	yes	no	1
6.In case of need,can you count on someone close to you?	yes	no	1
7.Do you regularly use a cane,a walker of a wheelchair to move about?	yes	no	1

PRISMA-7, the seven-question tool of the predictive accuracy of the Program on Research for Integrating Services of the Maintenance of Autonomy
