# Supplementary material for: Influence of frailty on short-term mortality in older patients with multiple trauma in the emergency department
Source: Eur J Trauma Emerg Surg. 2026 Jun 11;52(1):185. doi: 10.1007/s00068-026-03214-4 (PMC13260031; doi:10.1007/s00068-026-03214-4)
Supplement: Supplementary file 2 — Supplementary Material 2 [file 68_2026_3214_MOESM2_ESM.pdf]

Appendix2 Fifteen Variable Trauma Specifc Frailty Index (TSFI)

Item	Criterions		
**Comorbidities**			
Cancer History	Yes (1)	No (0)	
Coronary Heart Disease	MI (1)<br>Medication (0.25)	CABG (0.75)<br>None (0)	PCI (0.5)
Dementia	Severe (1)<br>No (0)	Moderate (0.5)	Mild (0.25)
**Daily Activities**			
Help with grooming	Yes (1)	No (0)	
Help managing money	Yes (1)	No (0)	
Help doing household work	Yes (1)	No (0)	
Help toileting	Yes (1)	No (0)	
Help walking	Wheelchair (1)<br>No (0)	Walker (0.75)	Cane (0.25)
**Health Attitude**			
Feel less useful	Most time (1)	Sometimes (0.5)	Never (0)
Feel sad	Most time (1)	Sometimes (0.5)	Never (0)
Feel effort to do everything	Most time (1)	Sometimes (0.5)	Never (0)
Falls	Most time (1)	Sometimes (0.5)	Never (0)
Feel lonely	Most time (1)	Sometimes (0.5)	Never (0)
**Function**			
Sexual active	Yes (0)	No (1)	
**Nutrition**			
Albumin	<3(1)	>3(0)	

The TSFI score is average score of the 15 items, ranging from 0 to 1. The higher the score, the weaker the body
